# Four new species of the genus *Cratospila* Foerster (Hymenopter, Braconidae, Alysiinae) from South Korea

**DOI:** 10.3897/zookeys.1022.62562

**Published:** 2021-03-08

**Authors:** JuHyeong Sohn, Cornelis van Achterberg*, Yun Jong Han, Hyojoong Kim

**Affiliations:** 1 Animal Systematics Lab., Department of Biology, Kunsan National University, Gunsan, 54150, South Korea Kunsan National University Gunsan South Korea; 2 State Key Laboratory of Rice Biology and Ministry of Agriculture / Key Lab of Agricultural Entomology, Institute of Insect Science, Zhejiang University, Hangzhou, 310058, China Zhejiang University Hangzhou China

**Keywords:** Alysiini, COI, Hymenoptera, new record, taxonomy, new species

## Abstract

The species of the genus *Cratospila* Foerster, 1863 (Braconidae, Alysiinae) from South Korea are revised, and the genus is recorded for the first time from South Korea. All four species are new to science, and *Cratospila albifera***sp. nov.**, *C. ejuncida***sp. nov.**, *C. luteocephala***sp. nov.**, and *C. syntoma***sp. nov.** are described and illustrated herein. In addition, COI has been sequenced of three species. A key to the Korean species is provided.

## Introduction

Alysiinae are a large subfamily of the family Braconidae and include two tribes; Alysiini and Dacnusini, with over 100 genera (Yu et al. 2016). Alysiinae occurs worldwide and contains over 2,440 valid species (Yu et al. 2016), of which 180 species in 21 genera are listed in the National Species List of South Korea (NIBR 2019). Alysiinae are known as koinobiont endoparasitoids of dipterous larvae, using their mandible to break open the puparium of the host. Some species of Alysiinae are commercially utilized for biological control (Abd-Rabou 2006).

The genus *Cratospila* Foerster, 1863, is a small, worldwide genus of Alysiinae, which includes 15 very similar species (Yu et al. 2016). Until now, four species are known from the Oriental region and two others are doubtfully known. Although Yaakop and Aman (2012) reported *C. circe* (Haliday, 1838) from Malaysia, this record most likely represents one of the very similar Oriental species of *Cratospila*. Also questionable is *C. curvabilis* Bhat, 1980 from India because its original description does not fit well with other species of *Cratospila*, and *C. curvabilis* probably belongs to another genus. Two other species occur in India and Bhutan, and Tobias (1990) described one species from Vietnam. In addition, Wharton (2002) described six species from Australia. Wu and Chen (1995) firstly reported a *Cratospila* species from China. Papp (1994) reported *C. circe* from North Korea, but without any details, and its record is doubtful because *C. circe* seems to be a Western Palaearctic species. Herein, we report for the first time the genus *Cratospila* from South Korea and include four newly discovered species. We present new morphological characters and the barcode region of the mitochondrial cytochrome c oxidase subunit I gene (COI) data of three of these new species. Descriptions, diagnoses, an identification key, and photographs of the diagnostic characters are provided.

## Materials and methods

Samples used in this study were collected at the following localities in South Korea: Inje-Gun, Gangwon (sweep net); National Arboretum of Korea, Gwangneung Forest, Pocheon-si, Soheul-eup, Gyeonggi-do (Malaise traps) and DMZ Botanical Garden, Mandae-ri, Haean-myeon, Yanggu-gun, Gangwon-do (Malaise traps). Sorting and preparation was done at the Department of Biology, Kunsan National University at Gunsan. For the identification of the genus Wharton et al. (1997) and Zhu et al. (2017) were used. The types are deposited in the Department of Biology, Kunsan National University at Gunsan (KSNU).

Morphological characters were observed with a Leica M205C stereo microscope. The Taxapad database (Yu et al. 2016) was used for references. For terminology used, see Wharton (2002) and van Achterberg (1993).

Extraction of DNA was done in ASL, KSNU. Whole genomic DNA was extracted from the specimens by using a DNeasy Blood & Tissue kit (QIAGEN, Inc., Dusseldorf, Germany) following the manufacturer’s protocol. In order to have complete voucher specimens after DNA extraction, non-destructive DNA extraction was performed with a slightly modified method from Favret (2005). A tissue lysis buffer with protease *K* solution was used to treat a leg at 55 °C for 12 h. The primers LCO-1490 (5'-GGTCAACAAATCATA AAGATATTGG-3') and HCO-2198 (5'-TAAACTTCAGGGTGACCAAAAAATCA-3') were used to amplify 658 bp as the partial front region of the COI and amplified by using AccuPowerH PCR PreMix (BIONEER, Corp., Daejeon) in 20 μl reaction mixtures containing 0.4 μM of each primer, 20 μM of the dNTPs, 20 μM of the MgCl_2_, and 0.05 μg of the genomic DNA template. The polymerase chain reaction (PCR) amplification was performed using a GS1 thermo-cycler (Gene Technologies, Ltd., U.K.) according to the following procedure: initial denaturation at 95 °C for 5 min, followed by 34 cycles at 94 °C for 35 sec; an annealing temperature of 48 °C for 25 sec; an extension at 72 °C for 45 sec, and a final extension at 72 °C for 5 min. The PCR products were visualized by electrophoresis on a 1.5% agarose gel. A single band was observed, purified using a QIAquick PCR purification kit (QIAGEN, Inc.), and then sequenced directly using an automated sequencer (ABI Prism 3730 XL DNA Analyzer) at Macrogen Inc. (Seoul, South Korea).

Sequence alignment were performed in MEGA version 7(Kumar et al. 2016) with ClustalW tool. The *P*-distance model was conducted using MEGA version 7.

## Results

A total of 605 bp of the COI fragment were sequenced from *Cratospila albifera* sp. nov. (GenBank accession no. MW376064), *C. luteocephala* sp. nov. (GenBank accession no. MW376065) and *C. syntoma* sp. nov. (GenBank accession no. MW376066). A pairwise distance was constructed by using the *P*-distance model with the option for pairwise deletion. As a result, the morphologically very similar *C. albifera* sp. nov. showed to differ genetically from *C. luteocephala* sp. nov. by 10% and from *C. syntoma* sp. nov. by 7%. In addition, *C. luteocephala* sp. nov. differed by 9% from *C. syntoma* sp. nov.

### Taxonomy

#### Cratospila

Taxon classificationAnimaliaHymenopteraBraconidae

Foerster, 1863

EA66AD56-7723-5BD0-B789-5E22A2403B2A

**Figure 1.**
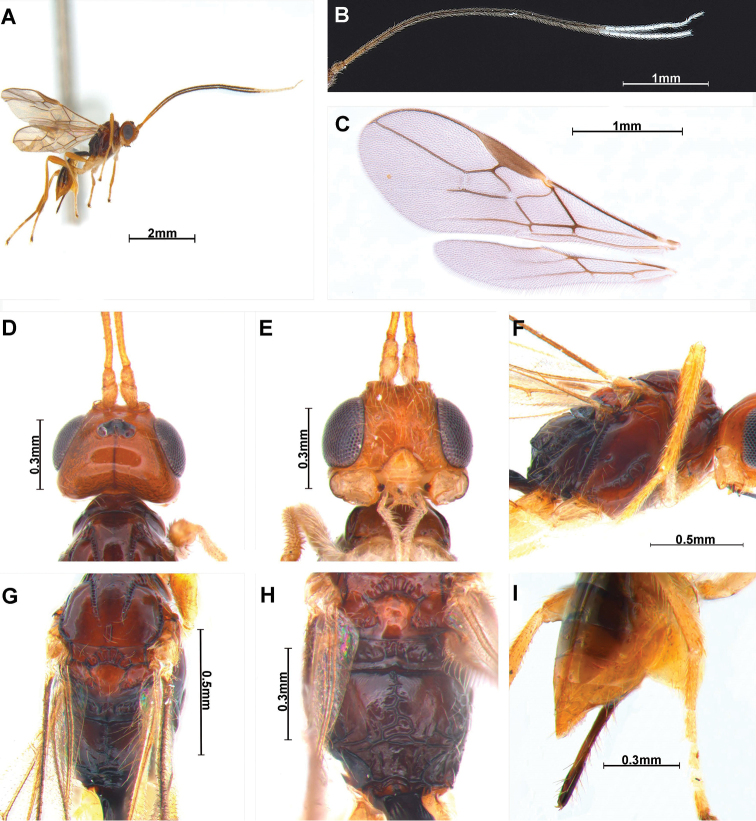
*Cratospila albifera* sp. nov. ♀. **A** body **B** antennae **C** wings **D** head, dorsal **E** head, frontal **F** mesosoma, lateral **G** mesosoma, dorsal **H** propodeum, dorsal **I** ovipositor sheath, lateral.

**Figure 2.**
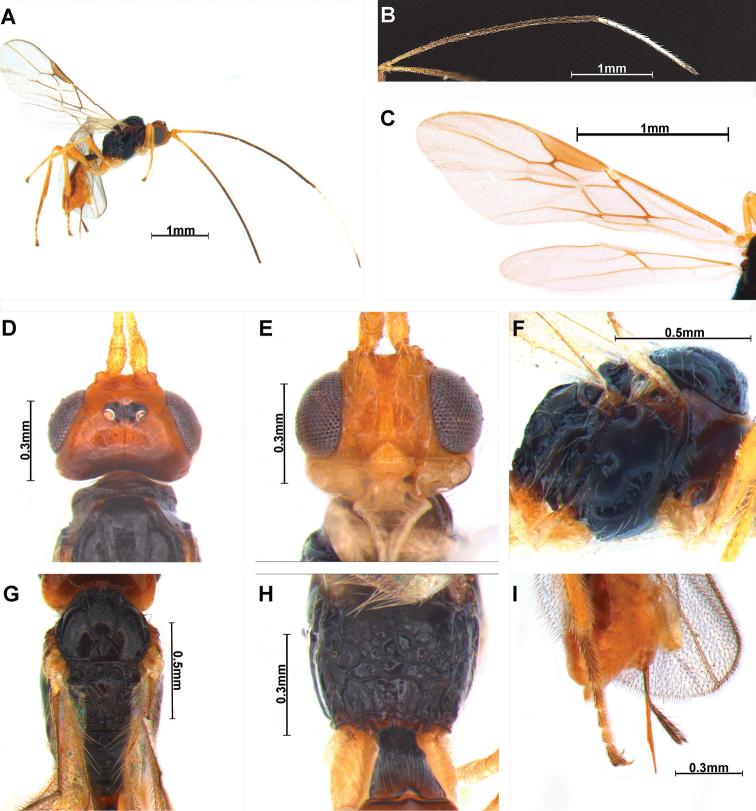
*Cratospila ejuncida* sp. nov. ♀ **A** body **B** antenna **C** wings **D** head, dorsal **E** head, frontal **F** mesosoma, lateral **G** mesosoma, dorsal **H** propodeum, dorsal **I** ovipositor and its sheath, lateral.

**Figure 3.**
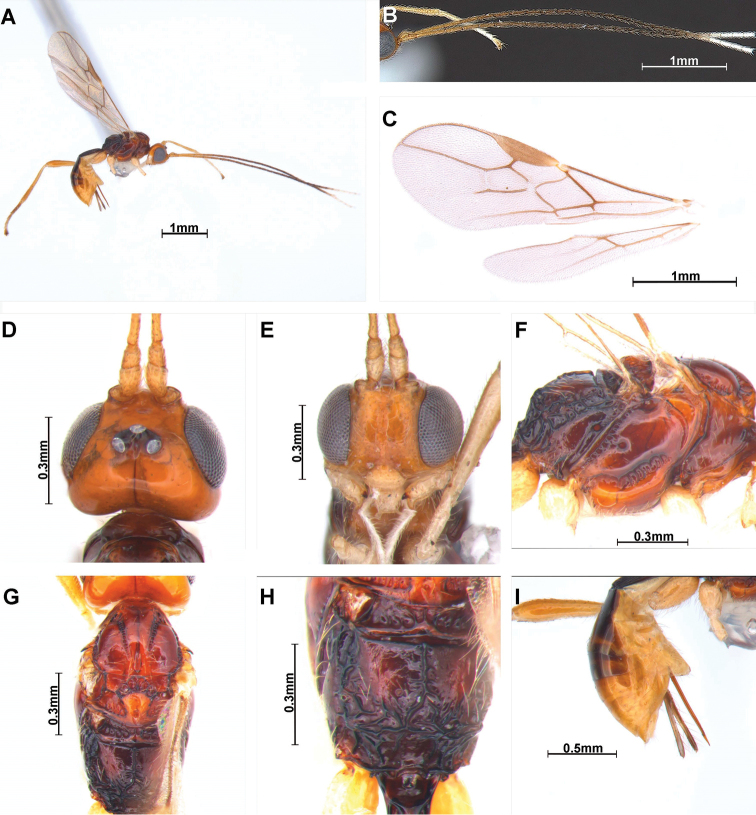
*Cratospila luteocephala* sp. nov. ♀ **A** body **B** antennae **C** wings **D** head, dorsal **E** head, frontal **F** mesosoma, lateral **G** mesosoma, dorsal **H** propodeum, dorsal **I** ovipositor and ovipositor sheath, lateral.

**Figure 4.**
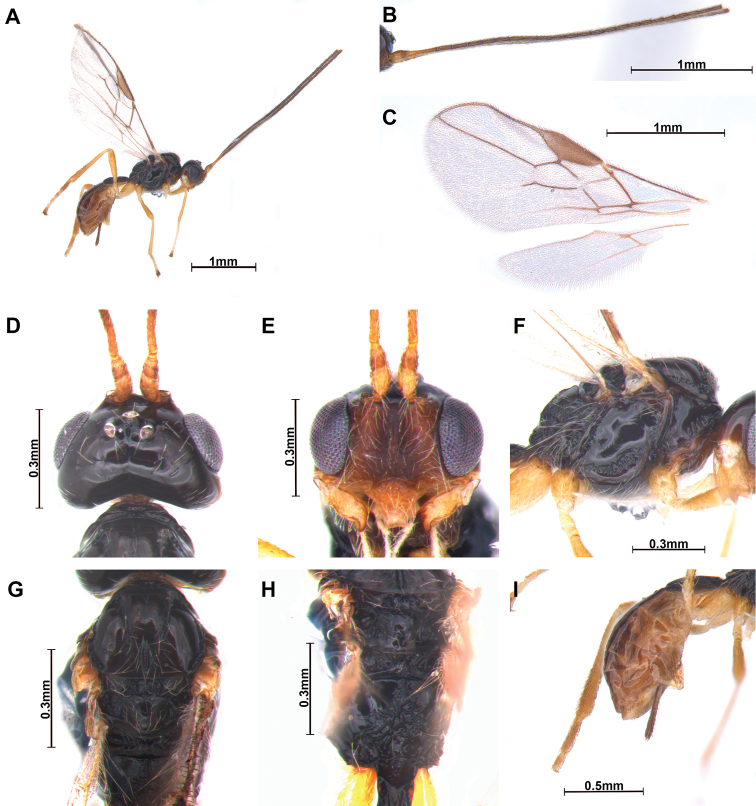
*Cratospila syntoma* sp. nov. ♀ **A** body **B** antennae **C** wings **D** head, dorsal **E** head, frontal **F** mesosoma, lateral **G** mesosoma, dorsal **H** propodeum, dorsal **I** metasoma and ovipositor sheath, lateral.

Figures 1 , 2 , 3 , 4

Cratospila Foerster, 1863: 265; Shenefelt. 1974: 985; Wharton 1980: 84; Tobias 1990; Belokobylskij 1998: 287; Zhu et al. 2017: 60. Type species (by monotypy): Alysia circe Haliday, 1838.
Hedylus Marshall, 1891: 14–15 (not Foerster, 1868); Papp 2009: 29–30 (as synonym of Cratospila because of the synonymising of both type species). Type species (by monotypy): Hedylus habilis Marshall, 1894 (examined; = Alysia circe Haliday, 1838).

##### Notes.

The genus can be identified by using the illustrated key to the Chinese genera of Alysiini by Zhu et al. (2017). The *Cratospila* species treated in this paper have the apical half of ♀ antenna with 8–13 white segments (unknown of *C. syntoma* sp. nov., but it has a largely yellowish-brown head, morphologically related to *C. ejuncida* sp. nov., and has according to the COI analysis a derived position compared with the other species); apex of antenna white, if dark brown then antennal white part 2.5–5.0 times as long as apical dark part of antenna. Papp (1994) reported *Cratospila circe* from North Korea, which is most likely a misidentification because this species is found so far only in the Western Palaearctic, and in the Eastern Palaearctic region there are several similar species. *Cratospila circe* can be separated from the new species described here by having the pale part of the female antenna either absent or present by a few whitish, greyish or ivory segments. and the pale part is 0.7–1.8 times as long as apical dark part of antenna.

##### Biology.

Rather small genus, of which the biology is unknown.

##### Distribution.

Cosmopolitan except Neotropical region.

### Key to species of *Cratospila* Foerster from South Korea

**Table:** 

1	Mesoscutum medio-posteriorly and scutellum reddish brown; head in dorsal view less transverse (Figs 1D, 3D) and yellowish brown; notauli on middle of mesoscutum comparatively coarsely crenulate (Figs 1G, 3G); pterostigma rather slender and narrowly yellow basally (Fig, 1C, 3C); vein r of fore wing 3–5 times longer than wide; vein 1-SR+M of fore wing slightly sinuate (Figs 1C, 3C); mesosoma 1.5–1.6 times longer than high in lateral view and anterior half of propodeum less sloping (Figs 1F, 3F); propodeum less extensively rugose medially (Fig, 1H, 3H); antennal sockets comparatively close to level of inner side of eyes (Figs 1E, 3E)	**2**
–	Mesoscutum medio-posteriorly and scutellum black; head in dorsal view more transverse (Figs 2D, 4D) and at least posteriorly darkened; notauli on middle of mesoscutum narrowly crenulate (Figs 2G, 4G); pterostigma rather robust and brown basally (Figs 2C, 4C); vein r of fore wing 1–2 times longer than wide; vein 1-SR+M of fore wing nearly straight (Figs 2C, 4C); mesosoma 1.4–1.5 times longer than high in lateral view and anterior half of propodeum largely sloping (Figs 2F, 4F); propodeum more extensively rugose medially (Figs 2H, 4H); antennal sockets more removed from level of inner side of eyes (Figs 2E, 4E)	**3**
2	Minimum width of face 0.9 times its height (measured from lower rim of antennal socket to upper medio-dorsal margin of clypeus; Fig. 3E); vein r of fore wing ca 3 times longer than wide; first subdiscal cell of fore wing ca 7.5 times longer than wide (Fig. 3C); [colour of apical antennal segments unknown]	***C. luteocephala* sp. nov.**
–	Minimum width of face 1.2 times its height (Fig. 1E); vein r of fore wing ca 5 times longer than wide; first subdiscal cell of fore wing ca 5.0 times longer than wide (Fig. 1C); [antenna of ♀ with ca 11 white segments, including apical segment]	***C. albifera* sp. nov.**
3	Second submarginal cell rather slender (vein 2-SR 1.8–1.9 times longer than vein 3-SR); vein r of fore wing twice as long as wide (Fig. 2C); first subdiscal cell of fore wing ca 8 times longer than wide; pedicellus entirely yellow; eye in dorsal view ca 2.1 times longer than temple (Fig. 2D); width of face 0.95 times its height; head (except posteriorly) yellowish brown (Fig. 2D); [antenna of ♀ with 10 or 11 white or ivory segments and apical segment dark brown, pale part 4.6 times longer than apical dark brown part]	***C. ejuncida* sp. nov.**
–	Second submarginal cell robust (vein 2-SR 1.4–1.5 times longer than vein 3-SR); vein r of fore wing approx. as long as wide (Fig. 4C); first subdiscal cell of fore wing ca 6 times longer than wide; pedicellus partly infuscated; eye in dorsal view ca 1.6 times longer than temple (Fig. 4D); width of face 1.1 times its height; head black dorsally (Fig. 4D); [colour of apical antennal segments unknown]	***C. syntoma* sp. nov.**

#### Cratospila albifera

Taxon classificationAnimaliaHymenopteraBraconidae

Sohn & van Achterberg sp. nov.

A6861A1A-FEDA-5745-966C-8F50258127F1

http://zoobank.org/8ABF4632-930C-431D-A637-C8A9949590CB

Figure 1

##### Type material.

***Holotype***, ♀ (NIBR), **South Korea**, National Arboretum of Korea, Gwangneung Forest, Pocheon-si, Soheul-eup, Gyeonggi-do, 37°45'32.2"N, 127°09'42"E, 16–30.IV.2018, Kim, Kim, Jo, Ki. GenBank accession no. MW376064. ***Paratype*.** 1♀, same data as holotype.

##### Comparative diagnosis.

Belongs to the group of *Cratospila* species together with *C. alboapicalis* Tobias, 1990, described from Vietnam in having the apical half of ♀ antenna with 8–13 white segments. However, in *C. alboapicalis* length of eye 4–5 times length of temple in dorsal view (1.9 times in the *C. albifera* sp. nov.) and antenna of ♀ with dark apical segments (only white segments in *C. albifera* sp. nov.). Differs from the very similar *C. luteocephala* sp. nov. by having the minimum width of face 1.2 times its height (0.9 times in *C. luteocephala* sp. nov.), vein r of fore wing ca 5 times longer than wide (ca 3 times), and first subdiscal cell of fore wing ca 5.0 times longer than wide (ca 7.5 times). COI sequence of *C. albifera* sp. nov. differs by 10% from that of *C. luteocephala* sp. nov. (Table 1).

**Table 1.** COI pairwise genetic distances between three new *Cratospila* species from South Korea.

	*Cratospila albifera*	*Cratospila luteocephala*	*Cratospila syntoma*
*Cratospila albifera*	0.000	0.101	0.093
*Cratospila luteocephala*	0.101	0.000	0.079
*Cratospila syntoma*	0.079	0.093	0.000

##### Description.

***Holotype***, ♀: length of body in lateral view 3.2 mm, length of antenna 4.6 mm, and length of fore wing 3.1 mm.

***Colour*:** body (Fig. 1A) brown, but head entirely orange-yellow; first tergite and mesonotum entirely reddish brown; antenna yellowish brown basally, medially dark brown, subapically white (11 flagellomeres); mandible pale orange.

***Head*** (Fig. 1D): width 1.3 times median length in dorsal view. Antenna (Fig. 1B) 1.4 times longer than body in female, 32-segmented. First flagellomere 2.0 times longer than second. Compounded eye slightly oval 1.1 times as long as wide in lateral view. Width of face (Fig. 1E) 1.2 times its height from ventral rim of antennal sockets to upper margin of clypeus. Eye in dorsal view 1.9 times as long as temple. Ocello-ocular line (OOL) 2.0 times longer than diameter of anterior ocellus; OOL: antero-posterior ocellar line (AOL) : postero-ocellar line (POL) = 11 : 3 : 6. Stemmaticum concave. Vertex smooth and polish with reddish brown line. Mandible with three teeth; second tooth narrow and sharp with dark brown tip. Maxillary palp approximately as long as mesosoma.

***Mesosoma*:** 1.5 times longer than wide in dorsal view. Mesosoma (Fig. 1G) with medio-posterior depression; notauli chain-shaped, completed but not reaching medio-posterior depression; scutellar sulcus with six carinae; metanotum sculptured; small basal bump on hind coxa. Propodeum (Fig. 1H) 0.5 times longer than wide, anterior half of propodeum smooth, posterior of median carina strongly wrinkled; precoxal sulcus (Fig. 1F) deep and distinct, consist of about seven grooves, lateral view of propodeum bent. Fore wing (Fig. 1C) 2.5 times as long as wide; pterostigma long and narrow, 3.9 times longer than wide; vein r of fore wing 4.7 times longer than wide; vein 2-SR slightly bent; vein 2-SR+M and r-m not sclerotized; vein 2-SR:vein r : vein 3-SR = 34 : 9 : 24; first subdiscal cell of fore wing ca 5.0 times longer than wide. Hind wing vein M+CU : vein 1-M = 66 : 5

***Leg*:** hind coxa compressed and grooved; hind coxa 2.8 times longer than hind trochanter; hind femur 0.9 times longer than hind tibia; hind tibia 0.8 times longer than hind tarsus.

***Metasoma*:** first tergite striate and narrow, 2.8 times longer than apical width and dark brown, T1:T2 = 59:23. Setose part of ovipositor sheath (Fig. 1I) 0.6 times as long as mesosoma, 0.5 times as long as hind tibia and with long setae.

**Male.** Unknown.

***Variation*.** Body length of female is 2.9–3.2 mm; length of the fore wing of female is 3.0–3.1 mm; Antenna 1.2–1.4 times longer than body in female, 27–32-segmented. First flagellomere 1.9–2.0 times longer than second; metasoma 2.7–2.8 times longer than apical width; setose part of ovipositor sheath 0.58–0.61 times as long as mesosoma, 0.46–0.51 times as long as hind tibia and with long setae.

##### Distribution.

South Korea.

##### Etymology.

Named after the white apex of the ♀ antenna: “*albifera*” is derived from “albus” (Latin for white) and “fero” (Latin for carry or bear).

#### Cratospila ejuncida

Taxon classificationAnimaliaHymenopteraBraconidae

Sohn & van Achterberg sp. nov.

A5D66BE6-FB80-5DA5-850E-B58F8F7A877A

http://zoobank.org/EC085A4F-BA86-4BB9-8442-60B6AA33F24B

Figure 2

##### Type material.

***Holotype***, ♀ (NIBR), **South Korea**, Inje-Gun, Bukmyeon, Hangyeri, 38°08'46.5"N, 128°15'47.5"E, 9–16. IX. 2017 (Malaise trap), J.H. Sohn.

##### Comparative diagnosis.

Belongs to the group of *Cratospila* species together with *C. alboapicalis* Tobias, 1990, described from Vietnam, in having the apical half of ♀ antenna with 8–13 white segments, and antenna of ♀ with dark apical part. In *Cratospila alboapicalis* length of eye 4–5 times length of temple in dorsal view (1.6 times in *Cratospila ejuncida* sp. nov.), vein m-cu of fore wing subinterstitial (distinctly antefurcal in *C. alboapicalis*), and notauli on middle of mesoscutum narrowly crenulate (coarser crenulate). Differs from the similar *C. syntoma* sp. nov. by having the second submarginal cell rather slender (vein 2-SR 1.8–1.9 times longer than vein 3-SR; 1.4–1.5 times in *C. syntoma* sp. nov.), vein r of fore wing twice as long as wide (approximately as long as wide), first subdiscal cell of fore wing ca 8 times longer than wide (6 times), pedicellus entirely yellow (partly infuscated), and eye in dorsal view ca 1.6 times longer than temple (ca 2.1 times).

##### Description.

***Holotype***, ♀: length of body in lateral view 2.5 mm (Fig. 2A), length of antenna 4.4 mm, and length of fore wing 2.5 mm.

***Colour*:** Head orange; antenna (except for two dark apical segments), with 11 flagellomeres white; mandible orange brown and apically dark brown. First tergite dark brown and mesonotum entirely black. Pedicellus entirely yellow.

***Head*** (Fig. 2D): width 1.2 times median length in dorsal view. Antenna (Fig. 2B) twice as long as body in female, 28 segmented. First flagellomere 1.8–1.9 times longer than second flagellomere. Compounded eye slightly oval 1.2 times as long as wide in lateral view. Width of face (Fig. 2E) 0.9–1.0 times its height from ventral rim of antennal sockets to upper margin of clypeus. Eye in dorsal view 1.6 times as long as temple. Ocello-ocular line 2.5 times longer than diameter of anterior ocellus; OOL : AOL : POL = 11 : 3 : 7. Stemmaticum concave. Vertex smooth and gloss with black line. Mandible with three teeth; first and third teeth smooth.

***Mesosoma*:** Mesosoma (Fig. 2G) 1.5–1.6 times longer than wide in dorsal view. Notauli on middle of mesoscutum narrowly crenulate, not reaching medio-posterior depression; scutellar sulcus with four carinae; metanotum sculptured; small bump in hind coxa adjacent to metapleuron. Propodeum (Fig. 2H) 0.6 times longer than width, more extensively rugose medially, lateral view of propodeum not bent; precoxal sulcus (Fig. 2F) is shallow and incomplete. Fore wing (Fig. 2C) 2.5 times as long as wide; pterostigma long and narrow, 4.2 times longer than wide; vein r of fore wing 1.9 times longer than wide; vein 2-SR slightly bent; vein 2-SR+M and r-m not sclerotized; vein 2-SR : vein r : vein 3-SR = 33 : 5 : 17; first subdiscal cell of fore wing ca 7.3 times longer than wide. Hind wing vein M+CU : vein 1-M = 69 : 4

***Leg*:** hind coxa compressed and grooved; hind coxa 1.5 times longer than hind trochanter; hind femur 0.6 times longer than hind tibia; hind tibia 1.01 times longer than hind tarsus.

***Metasoma*:** first tergite striate and narrow, brown, 2.5 times longer than apical width; T1:T2 = 41:23. Setose part of ovipositor sheath (Fig. 2I) 0.7 times as long as mesosoma, 0.5 times as long as hind tibia and with setae.

**Male.** Unknown.

##### Distribution.

South Korea.

##### Etymology.

Named after the relatively slender second submarginal cell of the fore wing: “*ejuncidus*” is Latin for slender.

#### Cratospila luteocephala

Taxon classificationAnimaliaHymenopteraBraconidae

Sohn & van Achterberg sp. nov.

73D70D6E-0B8F-57FD-9217-DD0D4005A950

http://zoobank.org/3055D636-AFE8-456A-A6FA-B40570050C00

Figure 3

##### Type material.

***Holotype***, ♀ (NIBR), **South Korea**, Inje-Gun, Bukmyeon, Hangyeri, 38°08'46.5"N, 128°15'47.5"E, 9–16. IX. 2017 (Malaise trap), J.H. Sohn. GenBank accession no. MW376065.

##### Comparative diagnosis.

Differs from other species of *Cratospila* by having the apical half of ♀ antenna with 8–13 white segments combined with a relatively wide face (1.2 times its height; 0.9–1.1 times in other species). Closely related to *C. albifera* sp. nov.; for differences, see they key above.

##### Description.

***Holotype***, ♀; length of body in lateral view 3.2 mm (Fig. 3A), length of antenna 4.2 mm (apex of antenna missing) and length of fore wing 2.9 mm.

***Colour*:** head (Fig. 3D) orange-yellow; with at least 4 flagellomeres of antenna white (apex of antenna missing); mandible whitish orange. First tergite dark brown and mesonotum entirely reddish brown.

***Head*:** width 1.4 times median length in dorsal view. Antenna (Fig. 3B) twice as long as body in female, 24 segmented (but apex of antenna missing). First flagellomere 1.8 times longer than second. Compounded eye slightly oval, 1.1 times as long as wide in lateral view. Width of face (Fig. 3E) 0.9 times its height from ventral rim of antennal sockets to upper margin of clypeus. Eye in dorsal view 1.7 times as long as temple. Ocello-ocular line 1.8 times longer than diameter of anterior ocellus; OOL : AOL : POL = 10 : 3 : 8. Stemmaticum concave. Vertex smooth, glossy, and with brown line. Mandible with three teeth; second tooth prominent, with black tip. Maxillary palp about equal length of mesosoma.

***Mesosoma*** (Fig. 3G): 1.5 times longer than wide in dorsal view. Mesoscutum with medio-posterior depression and setae near it; notauli on middle of mesoscutum, comparatively coarsely crenulate, not reaching medio-posterior depression; scutellar sulcus with six carinae; metanotum sculptured; small bump in hind coxa adjacent to metapleuron. Propodeum (Fig. 3H) 0.8 times longer than width, anterior half of propodeum less sloping; lateral view of propodeum is bent; precoxal sulcus (Fig. 3F) deep and distinct, consist of about nine grooves. Fore wing (Fig. 3C) 2.5 times as long as wide; pterostigma long and narrow, 4.1 times longer than wide; vein r of fore wing 3.2 times longer than wide; vein 2-SR slightly bent; vein 2-SR+M and r-m not sclerotized; vein 2-SR : vein r : vein 3-SR = 33 : 9 : 23; first subdiscal cell of fore wing ca 7.5 times longer than wide Hind wing vein M+CU : vein 1-M = 66 : 7

***Leg*:** hind coxa compressed and grooved; hind coxa 1.7 times longer than hind trochanter; hind femur 0.9 times longer than hind tibia; hind tibia 1.1 times longer than hind tarsus.

***Metasoma*:** first tergite striate and narrow, reddish brown, 2.8 times longer than apical width; T1:T2 = 59:24. Setose part of ovipositor sheath (Fig. 3I) 0.4 times as long as mesosoma, 0.5 times as long as hind tibia and with long setae (Fig. 2F).

**Male.** Unknown.

##### Distribution.

South Korea.

##### Etymology.

Named after its yellowish head: “*luteocephala*” is derived from “luteus” (Latin for yellow) and “cephalus” (Latin for head).

#### Cratospila syntoma

Taxon classificationAnimaliaHymenopteraBraconidae

Sohn & van Achterberg sp. nov.

C4576A34-2CE7-5A2E-A09D-808603411C42

http://zoobank.org/4F72B2F8-D2FD-4D00-BBDA-224067368CD5

Figure 4

##### Type material.

***Holotype***, ♀ (NIBR), **South Korea**, DMZ Botanical Garden, Mandae-ri, Haean-myeon, Yanggu-gun, Gangwon-do, 38°15'09.3"N, 128°06'40.6"E, 20.VI.–4.VII.2017, H.T. Shin, S.J. Kim. GenBank accession no. MW376066.

##### Comparative diagnosis.

Differs from other new species herein by the short vein r of the fore wing (ca as long as wide; 2–5 times in other species). Unfortunately, the antenna is incomplete but the COI analysis places it in the group of derived *Cratospila* species having the apical half of the ♀ antenna with 8–13 white segments (Table 1). Closely related to *C. ejuncida* sp. nov.; for differences, see the key above.

##### Description.

***Holotype***, ♀; length of body in lateral view 2.5 mm (Fig. 4A), length of antenna 2.8 mm (but apex of antenna missing) and length of fore wing 2.4 mm.

***Colour*:** head (Fig. 4D) entirely black; mandible yellowish brown. Antenna entirely dark brown (but apical segments missing). First tergite dark brown and mesonotum entirely black.

***Head*:** width 1.6 times median length in dorsal view. Antenna (Fig. 4B) 1.1 times longer than body in female, 23-segmented (apex of antenna missing). First flagellomere 1.7 times longer than second. Compounded eye slightly oval, 1.2 times as long as wide in lateral view. Width of face (Fig. 4E) 1.1 times its height from ventral rim of antennal sockets to upper margin of clypeus. Face with dense setae. Eye in dorsal view 1.9 times as long as temple. Ocello-ocular line 2.0 times longer than diameter of anterior ocellus; OOL : AOL : POL = 5 : 3 : 7. Stemmaticum concave. Mandible with three teeth; third tooth bent outside.

***Mesosoma*** (Fig. 4G): 1.9 times longer than wide in dorsal view with medio-posterior depression and setae near it; notauli on middle of mesoscutum narrowly crenulate, not reaching medio-posterior depression; scutellar sulcus with six carinae; metanotum sculptured; small bump in hind coxa adjacent to metapleuron; metapleuron with long setae. Propodeum (Fig. 4H) 0.8 times longer than width, more extensively rugose medially; lateral view of propodeum not bent; precoxal sulcus (Fig. 4F) completed with 10 grooves; scutellum with setae partially. Fore wing (Fig. 4C) 2.9 times as long as wide; pterostigma long and narrow, 3.2 times longer than wide; vein r of fore wing 1.5 times longer than wide; vein 2-SR slightly bent; vein 2-SR+M and r-m not sclerotized; vein 2-SR : vein r : vein 3-SR = 27 : 5 : 20; first subdiscal cell of fore wing ca 6 times longer than wide; second submarginal cell robust. Hind wing vein M+CU : vein 1-M = 39 : 4.

***Leg*:** hind coxa compressed and grooved; hind coxa 1.4 times longer than hind trochanter; hind femur 0.6 times longer than hind tibia; hind tibia 1.2 times longer than hind tarsus.

***Metasoma*:** first tergite striate and narrow, reddish brown, 2.7 times longer than apical width; T1:T2 = 45:19. Setose part of ovipositor sheath (Fig. 4I) 0.3 times as long as mesosoma, 0.4 times as long as hind tibia and with long setae.

**Male.** Unknown.

##### Distribution.

South Korea

##### Etymology.

Named after the short second submarginal cell of the fore wing: “*syntomus*” is Greek for shortened.

## Supplementary Material

XML Treatment for Cratospila

XML Treatment for Cratospila albifera

XML Treatment for Cratospila ejuncida

XML Treatment for Cratospila luteocephala

XML Treatment for Cratospila syntoma
